# Taurine bromamine (TauBr) - its role in immunity and new perspectives for clinical use

**DOI:** 10.1186/1423-0127-17-S1-S3

**Published:** 2010-08-24

**Authors:** Janusz Marcinkiewicz

**Affiliations:** 1 Department of Immunology, Jagiellonian University Medical College, Krakow, Poland

## Abstract

This review is an attempt to summarize our knowledge about taurine bromamine (TauBr) properties, its role in innate immunity and its therapeutic potential.

TauBr and taurine chloramine (TauCl) are major haloamines generated by eosinophils and neutrophils at a site of inflammation. Both haloamines share anti-inflammatory and anti-oxidant properties. TauBr, similarly to TauCl, decreases the production of proinflammatory mediators. Their anti-inflammatory and anti-oxidant activities are enhanced by their ability to induce the expression of heme oxygenase-1 (HO-1). TauCl is more stable than TauBr. On the other hand, only TauBr was found to be highly membrane-permeable showing stronger microbicidal activity than TauCl.

In the light of the anti-inflammatory and antimicrobial properties of TauBr we discuss its therapeutic potential in local treatment of inflammation, especially acne vulgaris, the most common inflammatory skin disorder. TauBr, at non-cytotoxic concentrations, is able to kill *Propionibacterium acnes*, the skin bacteria involved in pathogenesis of acne vulgaris.

As topical antibiotics used in the therapy of acne are associated with the emergence of resistant bacteria, topical TauBr seems to be a good candidate for an alternative therapy.

Recently, in a double blind trial, the efficacy of TauBr was compared with the efficacy of clindamycin, one of the most common topical antibiotics used in acne therapy. Comparable reduction of acne lesions was observed in the TauBr and clindamycin groups of patients with mild and moderate inflammatory facial acne vulgaris. We conclude that this pilot study supports our concept that TauBr can be used as a topical agent in the treatment of acne vulgaris, especially in patients who have already developed antibiotic resistance. Further studies are necessary to substantiate the more extended use of TauBr as an anti-inflammatory and anti-oxidant agent in human medicine.

## Introduction

Activated neutrophils and eosinophils generate a variety of reactive oxygen species (ROS). Hypochlorous acid (HOCl) and hypobromous acid (HOBr) are the major reactive oxidants generated by these cells at sites of inflammation. Both agents, components of the human innate immune system, exert strong microbicidal activity, but their excessive production leads to tissue damage [1,2]. Taurine, a sulfur-containing amino acid (2-aminoethane sulphonic acid), is the most abundant free amino acid in the leukocyte cytosol (30 mM) and is the major scavenger for both hypohalous acids, HOCl and HOBr [3]. The products of the reaction between taurine and HOBr or HOCl are taurine bromamine (TauBr, N-bromotaurine) or taurine chloramine (TauCl, N-chlorotaurine), respectively [4,5].

TauCl is considered the major haloamine produced *in vivo* by neutrophils. A large number of reports have shown a key role of TauCl in innate immunity and suggest its use in therapy of various topical infections as well as chronic inflammatory diseases [6]. In contrast, TauBr has attracted little attention because the extracellular concentration of bromide is at least 1,000-fold lower than that of chloride [2]. However, brominating intermediates such as HOBr and TauBr are potent antimicrobial agents *in vitro *[7,8].

In the present paper we discuss data showing anti-inflammatory and microbicidal properties of TauBr confirming its therapeutic potential. Finally, we demonstrate that TauBr may be a good candidate for a local treatment of skin inflammatory diseases, especially for acne vulgaris, a hypothesis supported by our recent pilot clinical study [9].

### In vivo generation of TauBr

Neutrophil myeloperoxidase (MPO) and eosinophil peroxidase (EPO) use hydrogen peroxide (H_2_O_2_) to oxidize halides and thiocyanate to their respective hypohalous acids. At plasma concentrations of halide (100 mM chloride; 20-100 μM bromide; <1 μM iodide) eosinophil peroxidase preferentially oxidizes bromide (Br^-^) to produce hypobromous acid, HOBr.

Br^-^ + H_2_O_2_ + H^+^ → HOBr + H_2_O

Recent studies have shown that HOBr may also be generated by the MPO-halide system of neutrophils.

HOCl + Br^-^ → HOBr + Cl^-^

HOCl and hypochlorite ion (OCl^-^) are therefore mainly produced by MPO, while HOBr and hypobromite (OBr^-^) are produced by both EPO and MPO [1,2].

HOBr, at physiological pH, reacts readily with amine compounds to form secondary oxidants such as mono-bromamines, di-bromamines and amino acid-derived aldehydes. These brominating agents, and HOBr in particular, contribute to innate immunity by virtue of having the ability to kill micro organisms but they may also damage host tissues and contribute to inflammatory tissue injury [10,11].

As taurine is the most abundant free amino acid in the leukocyte cytosol, TauBr is the major bromamine generated *in vivo* by eosinophil or neutrophil peroxidase. It is commonly accepted that taurine is the primary scavenger of overproduced or dislocated HOCl and HOBr. TauBr and TauCl are relatively long lived oxidants, and are less toxic than the corresponding hypohalous acids [4,5,12]. TauBr is therefore believed to protect cells against damage by HOBr [1,2]. However, in contrast to the large body of evidence demonstrating the role of TauCl in innate immunity, much less is known about microbicidal properties of TauBr [11-13].

### In vitro synthesis and detection of TauBr

To determine the function of TauBr, taurine mono-bromamine has been synthesized *in vitro* and used in a variety of experimental systems [8,10]. In our studies we have used the following protocol for synthesis and detection of TauBr *in vitro*.

**Figure 1:**
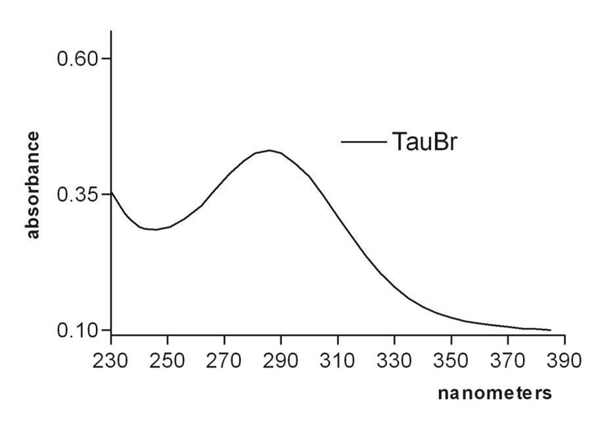
The UV absorption spectrum of taurine mono-bromamine (TauBr) λ_max_ = 288 nm.

TauBr preparation: HOBr/OBr^-^ was generated by mixing equimolar amounts of HOCl^ˉ^ and NaBr^ˉ^. Then, the product of this reaction was mixed with taurine. The mono-bromamine was obtained with a 10-fold excess of amine over the amount of HOBr/OBr^-^. Each preparation of TauBr was monitored by UV absorption spectra (λ=200 - 400 nm) to assure the authenticity of monobromamine (TauBr) (Fig. 1). Taurine bromamines (TauBr, TauBr_2_) have absorption spectra similar to those of chloramines, but shifted 36 nm towards longer wavelengths. The concentration of synthesized TauBr (taurine mono-bromamine) was determined using the molar extinction coefficient 415 M^-1^ cm^-1^ at A_288 _[1,8].

### TauBr and immune system

Our current knowledge concerning the physiological functions of TauBr is based mainly on experimental models investigating the effects of exogenous TauBr [9,10]. In studies with isolated leukocytes, evidence for the formation of endogenous brominating agents was obtained, but long lived bromamines were not detected in the medium. One may speculate that the reduction of HOBr^-^ and bromamines by H_2_O_2_^,^ produced by activated leukocytes, may account for these results. Indeed,* in vitro* studies from a number of laboratories show that TauBr, similarly to HOBr/Br^-^, is reduced by H_2_O_2_, resulting in the loss of oxidizing and brominating activity [2,10].

TauBr + H_2_O_2_ = Tau + H^+^ + Br^–^ + O_2_.

The ability of TauBr, to react and inactivate H_2_O_2 _[1] probably contributes to the reported “antioxidant” and anti-inflammatory properties of this compound. In addition, both TauBr and TauCl can induce the synthesis of heme oxygenase-1 (HO-1) [14], a stress-inducible enzyme, which also has antioxidant and anti-inflammatory capacity. TauBr, similarly to TauCl, is a powerful regulator of inflammation [10,15-17]. Both taurine haloamines exert anti-inflammatory properties by suppressing the production of such mediators as nitric oxide, PGE_2_, TNF-α, IL-6, IL-8, IL-12 and chemokines in both rodent and human leukocytes [10,15,18,19]. Studies investigating the mechanisms of action of TauCl have shown that it inhibits the activation of NF-κB, a potent signal transducer for inflammatory cytokines, by oxidation of IkB-α at Met^45^, and recent studies have extended these findings to TauBr [16].

**Figure 2:**
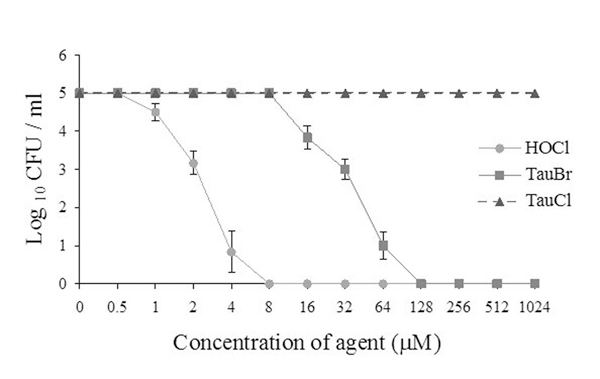
Bactericidal activity of TauBr, TauCl and HOCl against *E. coli *[10].

While the anti-inflammatory properties of endogenous TauCl and TauBr are well established, it is less clear whether these compounds also contribute to microbial killing. Our data (Fig. 2) show that TauBr has strong microbicidal activity comparable to HOCl at micro molar concentrations, while at these concentrations TauCl did not kill bacteria [8]. These results are in agreement with the studies of Gaut et al. [17], who found that addition of low concentration of bromide (1μM Br^-^) markedly increased bactericidal activity of the complete myeloperoxidase-H_2_O_2_–halide system. Therefore, one may speculate that physiological variations in Br^-^ concentration may amplify neutrophil bactericidal activity, by driving formation of HOBr and TauBr.

Importantly, TauBr, at bactericidal concentrations (< 200 μM) does not exert cytotoxic activity [10]. Moreover, it has been shown that TauBr at physiological concentrations is able to kill the schistosomula of *Schistosoma mansoni* confirming its role in the defence against parasites [11]. The contribution of chlorinating and brominating oxidants in pathogen killing and tissue injury will also depend on their interactions with other biologically active agents present at a site of inflammation. For example, TauBr may be neutralised by H_2_O_2_, as mentioned above [2,10].

### Therapeutic perspectives for TauBr

Is TauBr a good candidate for treatment of skin inflammatory diseases? A number of clinical studies have shown that TauCl may be useful for the treatment of various topical infections due to its combination of microbicidal and anti-inflammatory properties. However, in the majority of clinical trials TauCl was used at very high, non-physiological concentrations [6,20]. Much less is known concerning the therapeutic potential of TauBr.

**Figure 3:**
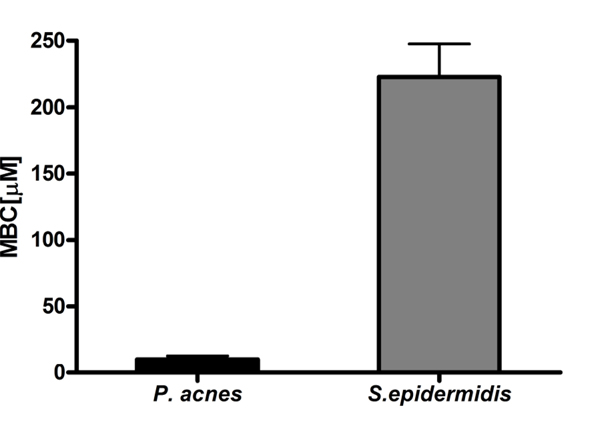
**Susceptibility of *P. acnes* and *S. epidermidis* to TauBr.** Data are expressed as MBC values (the concentrations of TauBr that cause 100% inhibition of bacteria growth) [9].

As discussed above, we have shown that TauBr *in vitro* has much stronger bactericidal activity than TauCl, with a potency which approaches that of HOCl, the most potent bactericidal agent of MPO-halide system [8]. Interestingly, susceptibility of *Propionibacterium acnes* (*P.acnes*) to TauBr appeared to be significantly higher than that of *Staphylococcus epidermidis (S.epidermidis)* (Fig. 3). Both species belong to the bacterial flora of the skin.

Importantly, TauBr killed all tested bacteria at non-cytotoxic, anti-inflammatory concentrations. From a clinical point of view these data strongly suggest that the therapeutic potential of TauBr may be similar or even better than that of TauCl.

Based on these studies of the biological properties and functions of taurine haloamines and on our studies demonstrating the selective antimicrobial activity of TauBr, we have examined the clinical efficacy of TauBr in the topical treatment of acne vulgaris, an inflammatory skin disease with bacterial etiology [9].

Acne vulgaris is the most common inflammatory skin disorder in adolescents and young adults [21]. Pathogenesis of acne is complex, involving multiple abnormalities of the pilosebaceous unit, including hyperkeratinisation, sebum production, bacterial proliferation and inflammation. One of the pathogenic factors of acne is *P. acnes*[22,23]. Topical antibacterial agents are an essential element of the armamentarium for the treatment of acne vulgaris [24,25]. As *P. acnes*, a potential pathogenic agent of acne, is extremely sensitive to TauBr, we hypothesized that TauBr may be a good candidate for the topical therapy for acne vulgaris, without the risk of inducing bacterial resistance [26,27].

In a double blind pilot study, the efficacy and safety of TauBr cream was evaluated [9]. Clindamycin gel, one of the most common topical agents in the treatment of acne vulgaris, was used as a control. Forty patients with mild to moderate inflammatory facial acne vulgaris were randomly treated with either TauBr or clindamycin. After 6 weeks, both treatments produced comparable beneficial results. More than 90% of patients improved clinically with a similar reduction in number of acne lesions (~65%), and with no side effects. Therefore, the results from this clinical pilot are in agreement with previous *in vitro* data and strongly suggest that TauBr could be considered a new therapeutic option in inflammatory acne.

## Conclusions

TauBr, the major bromamine generated at the site of inflammation, exerts both anti-inflammatory and anti-microbial properties, at non-cytotoxic concentrations. In addition, its ability to reduce other oxidants (e.g. H_2_O_2_) stresses the active role of TauBr in innate immunity. These biological properties make TauBr a good candidate for a topical treatment of inflammatory diseases. Further clinical investigations will be required to determine whether TauBr used in monotherapy or in combination with other agents may be a useful alternative treatment for acne vulgaris.

## Abbreviations

EPO: eosinophil peroxidase; HO-1: heme oxygenase; HOBr: hypobromous acid; HOCl: hypochlorous acid; H2O2: hydrogen peroxide; IL-6: interleukin -6; IL-8: interleukin-8; IL-12: interleukin-12; Met^45^: methionine^45^; MPO: neutrophil myeloperoxidase; NF-κB: nuclear factor- κB; OBr^-^: hypobromite ion; OCl^-^: hypochlorite ion; PGE_2_: prostaglandin E_2;_ ROS: reactive oxygen species; TauBr: taurine bromamine, N-bromotaurine; TauBr_2_: taurine dibromamine; TauCl: taurine chloramine, N-chlorotaurine; TNF-α: tumor necrosis factor- α.

## Competing interests

The author declares that he has no competing interests.
